# Critical Thinking and Metacognition: Pathways to Empathy and Psychological Well-Being

**DOI:** 10.3390/jintelligence13030034

**Published:** 2025-03-10

**Authors:** Miguel H. Guamanga, Carlos Saiz, Silvia F. Rivas, Patricia Morales Bueno

**Affiliations:** 1 Faculty of Human Sciences, Universidad Icesi, Cali 760000, Colombia; miguel.guamanga@u.icesi.edu.co; 2 Department of Basic Psychology, Psychobiology and Methodology of Behavioral Sciences, University of Salamanca, 37005 Salamanca, Spain; silviaferivas@usal.es; 3 Academic Department of Sciences, Pontificia Universidad Católica del Perú, Campus PUCP, 1801 Lima, Peru; pmorale@pucp.edu.pe

**Keywords:** critical thinking, metacognition, psychological well-being, empathy

## Abstract

This study examines the relationships between critical thinking, metacognition, psychological well-being, and empathy using structural equation modeling. The study sample consists of 155 university students from a higher education institution in Spain, who completed the PENCRISAL, the metacognitive abilities inventory, the Ryff psychological well-being scale, and the empathy quotient, which assess these psychological constructs. The results indicate that critical thinking has a direct positive effect on metacognition, which, in turn, is significantly associated with higher levels of psychological well-being and empathy. These findings reinforce the essential role of critical thinking in fostering cognitive self-regulation and socioemotional competencies. Furthermore, this study provides empirical evidence supporting the integration of critical thinking into educational programs, emphasizing its potential to enhance reflective thinking, emotional awareness, and interpersonal understanding.

## 1. Introduction: Exploring the Relationships Between Critical Thinking, Metacognition, Empathy, and Psychological Well-Being

In the fourth edition of *Thought and Knowledge: An Introduction to Critical Thinking*, Diane F. Halpern (2003) suggests a connection between critical thinking (CT) and empathy, arguing that solving interpersonal problems, similar to addressing other complex issues, require cognitive processes inherent to CT: accurately formulating the problem, generating alternative solutions, and selecting the most effective option. Interpersonal conflicts often involve ambiguous information, competing perspectives, and the need for reasoned judgment—elements that CT helps to resolve. Since empathy involves perspective-taking and emotional regulation, CT contributes to these processes by promoting analytical reasoning and reflective decision-making in social interactions. This perspective aligns with later theoretical contributions, such as those of Walters (1992, 1994) and Paul and Elder (2020), who also propose a relationship between CT and empathy. However, despite these shared conceptual foundations, the empirical exploration of this connection remains limited, as none of these works have led to a robust research tradition.

Beyond its connection to empathy, CT has also been identified as a catalyst for the development of metacognitive skills. According to Dwyer (2017), Halpern (1998) and Halpern and Dunn (2023), CT training facilitates the refinement of cognitive knowledge and cognitive self-regulation, both of which are essential components of metacognition. This is because systematic problem-solving and assertive decision-making—core processes within CT—require active reflection on one’s own cognitive processes, including their monitoring and adjustment when necessary. As a result, CT intervention programs not only strengthen an individual’s capacity for effective problem-solving, but also have the potential to contribute to an individuals’ psychological development and socioemotional well-being (Peng 2024; Su and Shum 2019).

Consequently, this study aims to investigate how CT, as an independent variable, directly influences metacognition. Furthermore, it explores the relationships between metacognition and other socioemotional domains, such as empathy and psychological well-being (PWB). Given that empathy involves both perspective-taking—the ability to understand others’ viewpoints—and emotional regulation—the capacity to manage affective responses in social interactions—these processes inherently require analytical reasoning and reflective decision-making. CT contributes to empathy by fostering deliberate perspective-shifting, evaluating alternative viewpoints, and regulating biases and emotional responses that may otherwise hinder objective understanding. By integrating these perspectives, this work seeks to provide a theoretical and empirical framework that elucidates these relationships and fosters practical applications in educational and social contexts.

Based on these objectives, the following section delineates the conceptual foundations of critical thinking, metacognition, empathy, and psychological well-being, aiming to articulate the theoretical bridges connecting these constructs.

### 1.1. Critical Thinking: Foundations and Practical Applications

Critical thinking theorists agree that CT is a teachable competency with a significant impact on various dimensions of knowledge and everyday life, both at the individual and societal levels. Its integrative nature encompasses a combination of skills, knowledge, dispositions, and actions that can be developed and applied across diverse contexts (Davies and Barnett 2015; Ennis 2018; Facione 1990; Paul and Elder 2020; Saiz 2020).

Recently, Steinert et al. (2022) have emphasized the relevance of CT for the effective functioning of democratic systems. By enabling citizens to access and critically process information while avoiding manipulation or misinformation, CT emerges as a crucial resource for informed decision-making that benefits both individuals and society. Its applications extend beyond academia, encompassing practical scenarios such as decision analysis and the resolution of everyday challenges. Additionally, CT depends not only on identifiable cognitive skills that can be developed, but also on non-cognitive factors, such as emotions, attitudes, and motivations (Brookfield 2007; Facione 1990; Halpern and Dunn 2023). These non-cognitive aspects, including open-mindedness, commitment to truth-seeking, and self-confidence in judgment, influence both the application and timing of cognitive skills.

The development and strengthening of CT require both cognitive skills—such as analysis, evaluation, and inference—and intellectual dispositions, which refer to the habitual inclination to apply these skills effectively in various contexts (Ennis 2015; Facione 1990). These dispositions include open-mindedness, intellectual humility, empathy, and a willingness to reconsider one’s beliefs, elements that influence how individuals engage in reasoning and decision-making (Hamby 2015; Perkins et al. 1993).

While this distinction may seem intuitive, it has been widely debated in the academic literature. Some scholars argue that CT instruction often prioritizes cognitive skills while neglecting these dispositional and affective components. A key critique of traditional CT instruction is that it adopts an overly formalistic and logic-based approach, potentially sidelining elements such as imagination and intuition, which also play a role in complex reasoning. Walters (1992, 1994) contends that this logicist view of CT fails to account for the nuanced ways in which reasoning operates in real-world scenarios.

In response, more recent proposals emphasize the role of emotions and empathy within CT, particularly when addressing the ethical and social dimensions of complex problems, such as interpersonal conflicts (Elder 1997; Gallo 1989; Lipman 2003; Paul and Elder 2006). By integrating cognitive skills with intellectual dispositions, these perspectives suggest that CT is not only a tool for logical analysis, but also a means for navigating complex social and moral dilemmas.

Grounded in these perspectives, this paper adopts a conceptualization of CT that emphasizes real-world applications, particularly those involving personal well-being and interpersonal relationships. Thus, in contrast to relating CT to purely academic or abstract tasks, this focus recognizes that CT often arises in everyday problem-solving scenarios where individuals navigate emotional, social, and practical challenges that directly impact their lives. Saiz’s proposal (Saiz 2024; Saiz and Rivas 2023; Guamanga et al. 2023) is particularly relevant in synthesizing these dimensions. According to Saiz, “to think critically is to arrive at the best explanation of a fact, phenomenon, or problem in order to know how to solve it effectively” (Saiz 2024, p. 19). This definition integrates cognitive components—such as analysis, inference, and evaluation—in the pursuit of the best explanation, along with non-cognitive elements like dispositions and motivations necessary for problem-solving (Ennis 2015). Furthermore, this definition expands the scope of CT beyond academic and professional boundaries, positioning it as a fundamental tool for addressing daily and interpersonal challenges with a practical and solution-oriented approach.

Given that CT involves systematic reasoning and problem-solving, this study examines how CT fosters the development of metacognitive skills, with a focus on enhancing cognitive self-regulation. Understanding this dynamic is key to clarifying the role of metacognition within the broader context of cognitive and socioemotional functioning.

### 1.2. Metacognition: The Mechanisms of Thought Monitoring and Regulation

Metacognition is present in all knowledge acquisition and plays a strategic role in problem-solving (Dwyer 2017; Flavell 1979; Halpern and Dunn 2023; Schraw and Moshman 1995). This implies that an individual must not only be aware of how cognitive processes work, but also be able to regulate and control these processes to optimize learning (Krathwohl 2002). In this sense, metacognition is directly related to the self-management and self-regulation of cognitive processes, manifesting itself in the ability to plan, monitor, and evaluate cognitive strategies (Flavell 1979; Schraw and Moshman 1995).

According to Flavell (1979), metacognition is defined as the ability to reflect on one’s own cognitive processes in order to consciously control and regulate them. This involves understanding how information is acquired, processed, and applied. Part of this knowledge can be deliberately accessed through a conscious search of memories to identify strategies that enhance learning and problem-solving, depending on the demands of a particular task and an individual’s cognitive abilities. Metacognition is distinguished primarily by two components: metacognitive knowledge and metacognitive control processes. Metacognitive knowledge includes three types: declarative (knowing the what about cognitive processes), procedural (knowing how to perform cognitive tasks), and conditional (knowing when and why to perform various cognitive actions). Metacognitive control processes involve the actions necessary to regulate and monitor thinking, and include the following components: planning, which involves selecting appropriate strategies and allocating resources efficiently before performing a task; monitoring, referring to the continuous evaluation of comprehension and performance during task execution; organization, which involves structuring information and processes in a coherent manner; debugging, to identify and correct errors in real time; and evaluation, focused on analyzing both the results obtained and the strategies used to determine their effectiveness (Schraw and Dennison 1994; Schraw and Moshman 1995).

In this context, metacognition is understood as the ability to reflect on, monitor, and regulate one’s own cognitive processes. This capacity allows for individuals to evaluate and adapt their learning and problem-solving strategies. Although metacognition is inherent, it can be further developed through targeted interventions. Specifically, when an intervention emphasizes CT, it often incorporates activities such as problem-solving and decision-making, which have been shown to significantly strengthen cognitive self-regulation skills (Dwyer 2017; Halpern and Dunn 2023). This promotes both the identification of effective strategies and their application to achieve optimal solutions and assertive decisions across diverse contexts, as could be the case in the socio-emotional domain, particularly in cognitive empathy.

### 1.3. Empathy: Cognitive and Affective Processes in Perspective-Taking

Empathy, according to various theories, comprises two main dimensions: cognitive empathy and affective empathy (Baron-Cohen 2022; Decety and Jackson 2004). The former refers to the ability to intellectually understand another person’s mental state, while the latter involves an emotional response to their feelings. Although these dimensions can theoretically be differentiated, in practice, they are often intertwined within the same human experience. For instance, understanding another person’s feelings (cognitive empathy) should typically elicit an appropriate emotional reaction (affective empathy). However, cases such as individuals with psychopathy or autism illustrate that it is possible to possess one dimension without the other.

In this paper, we adopt Simon Baron-Cohen’s perspective on empathy (Baron-Cohen 2003, 2022; Baron-Cohen et al. 2003; Baron-Cohen and Wheelwright 2004; Lawrence et al. 2004). Baron-Cohen (2022) conceptualizes empathy as a phenomenon that requires the decentering of personal focus to incorporate the perspective of others, a process he terms the double-minded approach. This decentering aligns with the concept of theory of mind, defined in psychology as the ability to attribute mental states to others—a fundamental skill for predicting behavior and responding appropriately. In practical terms, empathy enables a dual focus, allowing for individuals to balance their own perspective with that of others. In this context, Baron-Cohen describes empathy as a dimmer switch, capable of modulation based on an individual’s attentional engagement with others.

Based upon the understanding of empathy as a key socio-emotional competency, it is equally important to explore psychological well-being, which encompasses the broader dimensions of personal functioning.

### 1.4. Psychological Well-Being: Conceptualization and Key Dimensions

The multidimensional model proposed by Ryff (1989, 2014) and Ryff and Keyes (1995) conceptualizes PWB as a construct that extends beyond the emotional state of happiness or life satisfaction. Grounded in a eudaimonic philosophical approach, Ryff’s framework transcends traditional well-being indicators, such as life satisfaction or positive affect, and emphasizes the comprehensive functioning of the individual.

According to Ryff, PWB should not be confined to the subjective experience of feeling good or avoiding discomfort, but must be integrated into a broader perspective encompassing personal growth, the attainment of meaningful goals, and the effective management of life’s challenges. Within this model, Ryff delineates six core dimensions that serve as the foundational pillars of PWB: self-acceptance, environmental mastery, positive relationships with others, purpose in life, personal growth, and autonomy.

Self-acceptance pertains to a positive evaluation of oneself and one’s life, fostering emotional stability and a sense of personal fulfillment. Environmental mastery reflects the ability to effectively navigate and control external circumstances. Positive relationships emphasize the formation of meaningful bonds characterized by affection, trust, and mutual commitment. Purpose in life functions as a motivational force that guides actions toward the realization of significant goals. Personal growth involves openness to new experiences and overcoming challenges, contributing to self-actualization, while autonomy allows for greater alignment between personal values and actions, reinforcing independence and self-determination (Ryff 2014).

For the purposes of this paper, Ryff’s psychological well-being scale is considered to offer a robust conceptual foundation for identifying the potential contributions of CT interventions aimed at developing and enhancing metacognitive skills. Additionally, Ryff’s model serves as a practical operational framework, interwoven with essential processes such as decision-making and effective problem-solving, which are integral to well-rounded human development (Guamanga et al. 2024).

### 1.5. Theoretical Links Between Critical Thinking, Metacognition, Empathy, and Psychological Well-Being

The interplay between metacognition and CT reveals a strong conceptual link. Halpern and Dunn (2023) assert that effective decision-making requires individuals to consistently monitor and reflect on their reasoning processes. This reflection involves planning, assessing, and adjusting strategies to address problems effectively, which are hallmarks of metacognitive capacity. These processes are essential for higher-order reasoning, as they enable individuals to critically evaluate their own thought patterns and refine their decision-making strategies. CT, in turn, relies on these metacognitive processes while simultaneously refining them through deliberate and structured practice. Moreover, CT strengthens metacognition by engaging components such as planning, monitoring, error detection, organization, and evaluation. These elements are not only practiced during CT tasks, but are continually refined, promoting cognitive self-regulation and enhancing problem-solving efficacy (Arslan 2018; Kuhn 1999).

In an analogy, metacognition is to CT what the canvas is to a painting, and the artist’s technique is the metacognitive regulation. While metacognition is necessary in the acquisition of knowledge, as the canvas is to the artist, it is not sufficient. Technique is essential for the artist’s tone, traditions, and uniqueness to be captured on the canvas. In the same way, metacognitive regulation operates as problem-solving strategies. Theoretically, knowledge constitutes the what, the how, and the what for, but it is the implementation of strategies that leads to the materialization of knowledge. The interaction between CT and metacognition demonstrates their interconnected and mutually reinforcing nature, where CT provides a structured framework for reasoning, and metacognition facilitates the continuous monitoring, evaluation, and adjustment of these cognitive processes (Csíkos 2022).

Beyond its role in cognitive regulation, metacognition also plays a crucial role in socioemotional functioning, particularly in the development of empathy. Cognitive empathy, which requires a nuanced awareness of mental processes—both one’s own and those of others—depends on advanced cognitive self-regulation (Baron-Cohen 2022; Lawrence et al. 2004). This connection underscores the complexity of balancing an understanding of others’ emotions while effectively managing one’s own. This interplay demonstrates the essential role of metacognitive control in enabling empathy. The relationship between metacognition and empathy is another area of interest. Cognitive empathy, which requires a nuanced awareness of mental processes—both one’s own and those of others—depends on advanced cognitive self-regulation (Baron-Cohen 2022; Lawrence et al. 2004). This connection underscores the complexity of balancing an understanding of others’ emotions while effectively managing one’s own. This interplay demonstrates the essential role of metacognitive control in enabling empathy.

In addition to its influence on social cognition, metacognitive self-regulation plays a pivotal role in psychological well-being (PWB), as it allows for individuals to manage their thoughts, emotions, and behaviors in a constructive manner. The relationship between metacognition and PWB is grounded in the capacity for reflection and self-regulation, which are crucial for navigating life’s challenges. Key dimensions of PWB, such as self-acceptance, environmental mastery, and purpose in life, are strongly influenced by an individual’s ability to reflect on personal experiences and learn from them. Metacognition enables individuals to identify their cognitive strengths and weaknesses, refine their strategies, and execute action plans that align with their goals (Guamanga et al. 2024).

### 1.6. Empirical Evidence on the Relationships Between Critical Thinking, Metacognition, and Socioemotional Skills

The reviewed studies on the relationship between CT and empathy highlight a notable conceptual interaction, particularly between CT and cognitive empathy. Martingano and Konrath (2022) reported positive correlations between rational thinking indicators, such as the Need for Cognition Scale (NFC), and dimensions of cognitive empathy, such as perspective-taking (*r* = 0.33; *p* < .001), measured using Davis’ (1983) Interpersonal Reactivity Index (IRI). Conversely, when examining rational performance using the Cognitive Reflection Task (CRT), negative correlations were observed with emotional empathy dimensions, such as Personal Distress (PD; *r* = −0.13, *p* = .006). These findings suggest that cognitive empathy is closely aligned with deliberative and rational processes, whereas emotional empathy tends to be more automatic and, at times, may even conflict with rational thinking.

Lombard et al. (2020) found that critical thinking (CT) and cognitive empathy are linked through the concept of decentering, which fosters recognition of diverse perspectives and values. Zhao et al. (2024) further proposed that CT played a key role in shaping the relationship between multicultural experiences and empathy, with its influence being more prominent in fostering cognitive empathy than emotional empathy. This underscores the reliance of cognitive empathy on analytical processes, while emotional empathy remains tied to affective and automatic responses.

Other studies, such as Heiney et al. (2019), have explored the integration of these skills within specific educational contexts, demonstrating improvements in students. Similarly, Varghese (2018) proposed that CT, by challenging dominant ideologies, and empathy, by fostering social cohesion, can underpin educational practices aimed at promoting social justice

In addition to empathy, emotional intelligence (EI) has also been identified as a key socioemotional factor influencing psychological well-being (PWB). A substantial body of research demonstrates a robust conceptual and empirical link between EI and PWB. For instance, Augusto-Landa et al. (2011) reported that emotional clarity and emotional regulation are significant predictors of eudaimonic well-being, particularly in dimensions such as purpose in life (*β* = 0.34) and self-acceptance (*β* = 0.46). Additionally, emotional regulation exerts an indirect influence through optimism (*β* = 0.63) and pessimism (*β* = −0.23), together explaining 60% of the variance in PWB. These results underscore the importance of emotional clarity as a key resource for managing emotions and optimizing cognitive processes. They also open new pathways for exploring how other dimensions of EI, such as resilience and stress management, affect psychological and relational well-being.

In this same line of research, Burrus et al. (2012) emphasized that managing emotions is crucial for fostering resilience and coping with stress, both of which significantly contribute to overall well-being. The research, assessed using the Situational Test of Emotion Management (STEM), demonstrated significant correlations between emotional management and eudaimonic well-being (*r* = 0.54, *p* < .001). Furthermore, emotional management was positively associated with hedonic well-being, such as positive affect, and was negatively correlated with negative affect (*r* = −0.46 to −0.18). These results show that individuals with higher levels of EI experience greater emotional balance, especially in activities like work (*r* = 0.44) and exercise (*r* = 0.34). Similarly, Altaras Dimitrijević et al. (2018) confirmed that emotional regulation was the strongest predictor of PWB, surpassing academic intelligence. Emotional management presented a direct association with PWB (*β* = 0.32, *p* < .001), adding 8% to its explained variance.

Guerra-Bustamante et al. (2019) strengthened this evidence, focusing on adolescents. They found that emotional clarity and repair significantly increased subjective happiness. Adolescents with high emotional clarity were 5.6 times more likely to report high levels of happiness (*OR* = 5.64, *p* < .05). This probability rose to 12 times for those with strong emotional repair skills (*OR* = 12.13, *p* < .05). However, excessive emotional mindfulness was found to be counterproductive, suggesting that balance is key.

The interaction between CT and PWB presents a more complex dynamic. While EI and PWB primarily align through emotional clarity and regulation, CT operates through distinct yet complementary cognitive mechanisms. Su and Shum (2019) observed that CT impacts PWB indirectly through mediators like cognitive distortions, moderated by mindfulness. In conditions of low mindfulness, CT was significantly associated with cognitive distortions (*β* = 0.51, *p* = .01), which, in turn, predicted higher levels of psychological distress (*β* = 0.30, *p* < .05). Conversely, high mindfulness mitigated these effects, reducing the negative impact of CT on distress.

This suggests that, while CT primarily engages analytical and reflective reasoning, its effectiveness in promoting PWB is influenced by how individuals regulate their emotions. Managing emotions, particularly through emotional clarity, plays a crucial role in reducing cognitive distortions and enhancing the capacity for deliberate decision-making. In this way, emotional regulation acts as a supporting mechanism that enables individuals to apply CT more effectively in emotionally charged situations, reinforcing the connection between cognitive and affective processes.

Peng (2024) expanded this line of inquiry by exploring how CT and cultural intelligence jointly predicted dimensions of eudaimonic well-being, such as purpose in life and self-acceptance. Peng reported significant correlations between CT and dimensions like purpose in life (*r* = 0.73, *p* < .01), and between cultural intelligence and well-being (*r* = 0.83, *p* < .01). Among the factors contributing to cultural intelligence, cultural metacognition stood out (*r* = 0.72, *p* < .01). Together, they explained 45% of the variance, with CT contributing 47.3% (*β* = 0.47, *p* < .001) and PWB contributing 27.1% (*β* = 0.27, *p* < .01). These findings highlight the complementary role of cognitive skills and personal development in fostering academic and personal success.

From a theoretical perspective, integrating the cognitive domain of CT with the affective domain of socioemotional competencies enhances the interdisciplinary framework by establishing connections between analytical reasoning and emotional regulation. Pedagogically, validating this connection supports transformative learning strategies tailored to the demands of a globalized, complex environment. Preparing students to critically analyze problems, regulate their emotions, and maintain a clear sense of purpose is an educational priority that transcends traditional classroom boundaries.

The reviewed literature provides a robust foundation for understanding the dynamic relationships among CT, metacognition, empathy, and PWB. Studies highlight the conceptual relationship between CT and cognitive empathy, particularly through processes such as perspective-taking, where rational and analytical thinking facilitates the understanding of diverse perspectives and values (Martingano and Konrath 2022; Lombard et al. 2020). Additionally, CT has been linked to PWB through its capacity to promote reflective and deliberate decision-making, contributing to outcomes such as purpose in life and self-acceptance (Peng 2024; Su and Shum 2019). Collectively, these findings underscore the cross-disciplinary importance of CT and metacognition in fostering socioemotional development and resilience, particularly within educational contexts (Varghese 2018; Zhao et al. 2024).

With this empirical foundation as a basis, it becomes essential to adopt a methodological approach capable of capturing the complexity of these interrelated constructs and their dynamic interactions. Given the complex and multidimensional nature of the constructs involved, this study employs structural equation modeling (SEM). SEM is particularly well-suited for this analysis, as it enables the simultaneous estimation of multiple relationships among latent variables while accounting for measurement error, thereby providing a more accurate and reliable representation of the theoretical model (Kline 2023; Marôco 2014). By integrating measurement and structural components within a single analytical framework, SEM is expected to provide robust statistical evidence for the hypothesized relationships, potentially facilitating a comprehensive understanding of the dynamic interactions between cognitive and socioemotional processes.

According to the theoretical model designed, we propose the following hypotheses:

**H1.**  *There is a significant positive relationship between CT and metacognition, such that students with higher CT skills demonstrate higher levels of metacognition.*

**H2.**  *Metacognition has a statistically significant positive direct effect on both empathy and PWB.*

## 2. Materials and Methods

### 2.1. Participants

A total of 155 students (*M* = 19.0, *SD* = 2.20) from a university in the northeastern region of Spain participated in this study. Of these, 88.9% were women and 11.1% were men, all enrolled in either the first or final year of an undergraduate psychology program.

### 2.2. Instruments

The following tests were used for data collection.

#### 2.2.1. Metacognitive Abilities Inventory

The Metacognitive Abilities Inventory (Schraw and Dennison 1994), validated in a Spanish-speaking sample by Huertas Bustos et al. (2014), is a psychometric tool designed to assess students’ metacognitive abilities in terms of two main components: knowledge of cognition and regulation of cognition. Each of these components encompasses several subprocesses, which Schraw and Moshman (1995) identify as core dimensions of metacognition. Specifically, metacognitive knowledge consists of three main types—declarative, procedural, and conditional—while the regulation of cognition includes planning, organization, monitoring, debugging (i.e., error correction), and evaluation of one’s own cognitive processes. The inventory comprises 52 items and demonstrated high reliability, with a Cronbach’s alpha of 0.94 in its validation.

#### 2.2.2. Empathy Quotient (EQ)

The Empathy Quotient (EQ), created by Baron-Cohen and his team, is a self-assessment questionnaire designed to assess individual variations in empathy among adults of average intelligence (Baron-Cohen and Wheelwright 2004). The EQ includes 40 items focused on empathy and 20 additional items that act as control items but which are not used to calculate the empathy score. The assessment instrument is composed of three key factors: Cognitive Empathy (CE), which measures the ability to perceive and understand emotional and mental states of others; Emotional Reactivity (ER), which assesses the tendency to have emotional responses to the mental states of others; and Social Skills (SS), which examines the intuitive ability to interact in social situations (Baron-Cohen and Wheelwright 2004; Redondo and Herrero-Fernández 2018). The Empathy Quotient (EQ) demonstrated high internal consistency, with a Cronbach’s alpha of 0.92, confirming its reliability.

#### 2.2.3. PENCRISAL

The PENCRISAL (Rivas and Saiz 2012; Saiz and Rivas 2008) consists of 35 items, each representing a problem situation, and is structured into 5 factors: deductive, inductive, and practical reasoning, decision-making, and problem-solving. The PENCRISAL test originated from the HCTA-Test (Halpern 2012), and its principles include the use of everyday situations, different domains, an open-ended response format, and the use of single-answer questions. For the validation process, studies were conducted in 715 Spanish adults aged between 18 and 53 years. The results show reliability in internal consistency (Cronbach’s α = 0.63) and in test–retest reliability (*r* = 0.79).

#### 2.2.4. Ryff’s Psychological Well-Being Scale

The Spanish adaptation of the Ryff psychological well-being scale by Díaz et al. (2006), consisting of 39 items, was used. The results on internal consistency and construct validity indicated that the scales demonstrated adequate reliability, with Cronbach’s alpha values ranging from 0.70 to 0.83 across dimensions. However, some studies have reported difficulties in replicating the six-factor model across different contexts and populations (Abbott et al. 2010). Consequently, based on prior research examining the relationship between critical thinking and psychological well-being (Guamanga et al. 2024), this study focuses on three dimensions: self-acceptance, purpose in life, and environmental mastery.

### 2.3. Intervention Program

For this research, the ARDESOS-DIAPROVE program was implemented. This program is an educational intervention designed to enhance critical thinking skills in areas such as argumentation, decision-making, and problem-solving (Saiz and Rivas 2011; Saiz 2020; Rivas and Saiz 2023; Guamanga et al. 2023). It was delivered in person over one semester (approximately 16 weeks), comprising about 58 h of instruction, and was facilitated by instructors with specialized training in CT. Throughout the sessions, participants engaged in problem-based learning (PBL) activities and employed metacognitive strategies, emphasizing (a) the identification and management of cognitive biases, (b) the combined use of factual information and deductive reasoning, and (c) the application of hypothesis-refutation processes when evidence did not support specific claims.

### 2.4. Procedures

To collect data, digital questionnaires were administered at the beginning and conclusion of the first-year and fourth-year CT courses. Participants received detailed instructions and were assured of the confidentiality of their responses, thereby ensuring data integrity and reliability. For the present study, only the final assessment data were used in the statistical analyses, ensuring that the results reflect the participants’ most developed CT competencies. Only those who completed all questionnaires were included in the final analysis, preserving the rigor and validity of the results.

### 2.5. Data Analysis

Data were analyzed using the IBM-SPSS Statistics v.28. The causal model was carried out using the maximum likelihood estimator available in AMOS 25.0 (Arbuckle 2016). To assess the models’ goodness-of-fit, the following indicators were used: the Chi-square by degrees of freedom ratio (χ^2^/df), the Comparative Fit Index (CFI), and the Root Mean Square Error of Approximation (RMSEA). Values below 5 for χ^2^/df, above 0.90 for CFI and below 0.08 for RMSEA indicate an acceptable model data fit (Kline 2023; Marôco 2014).

## 3. Results

Table 1 presents the correlations between the variables of CT, empathy, metacognition, and PWB, as well as all of their dimensions. The data indicate that there are significant relationships of varying magnitude. The strongest correlation is observed between metacognition and PWB (*r* = 0.59, *p* < .01), suggesting a moderate to high relationship between these variables. Conversely, the weakest correlation was found between CT and empathy (*r* = 0.11, not significant), reflecting a weak and non-significant relationship. Additionally, the correlations between CT and metacognition (*r* = 0.26, *p* < .01) and between CT and PWB (*r* = 0.21, *p* < .01) were significant, although of lesser magnitude compared to other relationships.

In Table 1, positive and significant correlations were observed between several dimensions of CT and the social skills (SS) dimension of empathy. The correlation between CT general and SS is *r* = 0.27, *p* < .01; between decision-making (DM) and SS, *r* = 0.25, *p* < .01; and for deductive reasoning (DR) with SS, *r* = 0.24, *p* < .01. Inductive reasoning (IR) also shows a significant correlation with SS (*r* = 0.17, *p* < .05). CT showed a positive and significant correlation with the overall psychological well-being (PWB) score (*r* = 0.21, *p* < .01), as well as with specific dimensions: self-acceptance (SA; *r* = 0.21, *p* < .01), environmental mastery (EM; *r* = 0.20, *p* < .05), and purpose in life (PL; *r* = 0.20, *p* < .05).

Furthermore, Table 1 shows positive and significant correlations between metacognition and empathy (*r* = 0.36, *p* < .01) and between metacognition and PWB (*r* = 0.59, *p* < .01), indicating moderate-to-strong associations across these cognitive and socioemotional constructs.

The findings indicate positive, albeit modest to moderate, associations between metacognition and both psychological well-being (PWB) and empathy, underscoring the relevance of metacognition as an independent cognitive construct across diverse psychological domains. Additionally, the total score of CT shows significant positive, albeit moderate, correlations with both metacognition and PWB. To analyze these relationships, a Structural Equation Model (SEM) was implemented, capturing associations among the constructs without presupposing mediating or moderating effects.

The SEM demonstrated an adequate model fit, as indicated by multiple goodness-of-fit indices aligning with the thresholds established by Marôco (2014). The chi-square to degrees of freedom ratio (*χ*^2^/df) was 1.484, reflecting a good fit. The Comparative Fit Index (CFI) reached 0.938, exceeding the conventional threshold of 0.90 for a good fit and approaching the benchmark of 0.95 for an excellent fit. Furthermore, the Root Mean Square Error of Approximation (RMSEA) was 0.056, with a 90% confidence interval of [0.040–0.071], which falls within the acceptable fit range (0.05–0.08) and nears the threshold for a close fit (<0.05). Collectively, these indices provide adequate evidence supporting the model’s statistical validity and its alignment with the underlying theoretical framework.

The improved model fit was achieved by incorporating correlations between specific residual errors within the metacognition construct, as suggested by modification indices derived from a prior model. A detailed review of the associated items revealed their alignment with the domains of planning, monitoring, and evaluation, as operationalized in the Metacognitive Awareness Inventory (Schraw and Dennison 1994). These items exhibit overlap in their conceptual focus, as they all address critical aspects of metacognitive regulation. This shared foundation likely accounts for their residual variance, which is not fully explained by the latent variable.

The decision to model covariances between these residuals aligns with established practices in structural equation modeling, particularly when supported by both statistical evidence and theoretical justification (Kline 2023). Such covariances do not imply causality, but rather account for unmodeled interdependencies among observed variables, thereby enhancing the precision of the overall model. This adjustment ensures that the model provides an accurate representation of the relationships among constructs while preserving its theoretical integrity.

**Figure 1:**
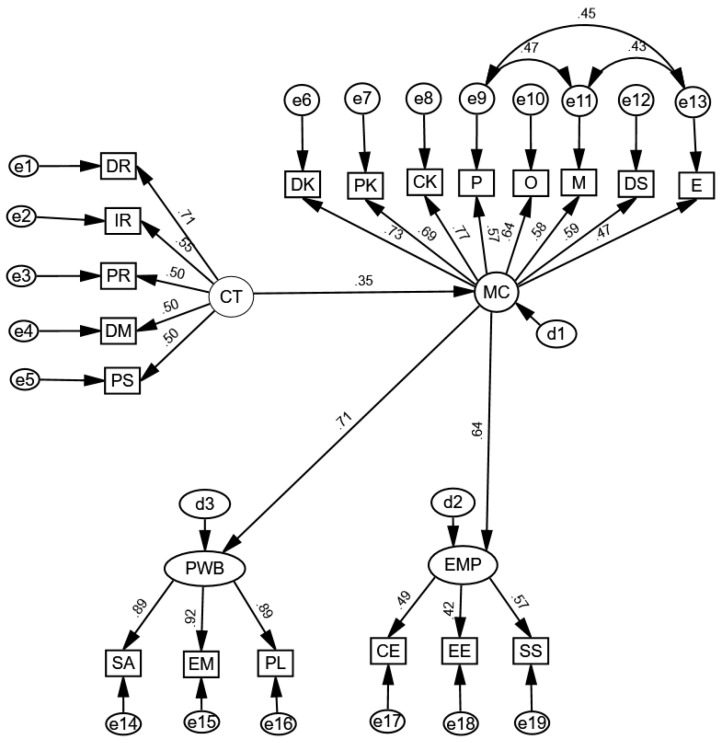
Path diagram. Structural equation model. CT: critical thinking, DR: deductive reasoning, IR: inductive reasoning, PR: practical reasoning, DM: decision-making, PS: problem-solving; EMP: empathy, CE: cognitive empathy, EE: emotional reactivity, SS: social skills; MC: metacognition, DK: declarative knowledge, PK: procedural knowledge, CK: conditional knowledge, P: planning, O: organization, M: monitoring, DS: debugging strategies, E: evaluation; PWB: psychological well-being, SA: self-acceptance, EM: environmental mastery, PL: purpose in life.

Figure 1 illustrates the final structural model. The structural model captures the direct effect of CT on metacognition, alongside the role of metacognition in shaping both empathy and PWB.

Table 2 presents the results of structural equation modeling (SEM), which examined the hypothesized relationships among the study variables. The path from CT to metacognition yielded an unstandardized estimate of 0.67 (*SE* = 0.23), with a critical ratio (*CR*) of 2.99, *p* = .003, indicating a moderate effect. This relationship is further supported by the standardized coefficient (*β* = 0.35). The path from metacognition to PWB demonstrated a stronger association, with an unstandardized estimate of 1.54 (*SE* = 0.20), a *CR* of 7.62, and *p* < .001, corresponding with a strong effect (*β* = 0.71). Similarly, the path from metacognition to empathy was statistically significant, producing an unstandardized estimate of 0.39 (*SE* = 0.09), a *CR* of 4.38, and *p* < .001, with a standardized coefficient of *β* = 0.64, indicating a strong relationship.

## 4. Discussion

CT emerges in this study as a key factor in strengthening metacognitive processes, which are significantly associated with psychological well-being (PWB) and empathy. The results support the proposed hypotheses, demonstrating that interventions focused on developing CT, particularly those targeting problem-solving and decision-making, substantially enhance metacognitive self-regulation. Additionally, metacognition has a significant impact on socio-emotional skills, such as empathy, and on personal development dimensions related to PWB, reinforcing its role in comprehensive human development. These findings align with previous research highlighting CT as both a fundamental cognitive skill and a resource for enhancing personal and socio-emotional functioning (Saiz 2020; Saiz and Rivas 2023). The structural equation model demonstrated strong fit indices (CFI = 0.938; RMSEA = 0.056), corroborating the robustness of these associations.

Regarding the first hypothesis, the results confirm that CT positively influences metacognition. This supports the view that CT fosters reflection and cognitive self-regulation. Consistent with prior studies, CT training improves metacognitive awareness by encouraging individuals to monitor, evaluate, and adjust their cognitive processes (Arslan 2018; Dwyer 2017; Halpern and Dunn 2023; Kuhn 1999; Saiz 2020). The ability to critically analyze situations, draw inferences, and evaluate decisions is closely linked to metacognitive regulation, underscoring the importance of integrating CT into educational programs to develop problem-solving skills across contexts.

With respect to the second hypothesis, the findings show that metacognition has a positive and significant effect on both PWB and empathy. This suggests that metacognitive skills are crucial for psychological well-being and interpersonal functioning. The association between metacognition and PWB aligns with research indicating that self-reflection and cognitive regulation contribute to self-acceptance, environmental mastery, and a clear sense of purpose (Augusto-Landa et al. 2011). The ability to evaluate personal experiences, regulate emotions, and adapt to life’s challenges is fundamental to well-being.

Similarly, the relationship between metacognition and empathy highlights the cognitive dimension of empathy, particularly perspective-taking and understanding others’ mental states. This aligns with Baron-Cohen’s (2022) assertion that cognitive empathy relies on the ability to understand and predict others’ thoughts and emotions, a process dependent on developed metacognitive skills. Our findings support evidence that individuals with stronger cognitive self-regulation are better equipped to interpret and respond appropriately to others’ emotions, enhancing social interactions (Lawrence et al. 2004; Martingano and Konrath 2022). From an applied perspective, these findings have significant educational implications. Integrating CT into curricula not only enhances cognitive competencies, but also strengthens metacognitive skills essential for emotional self-regulation and personal well-being (Rivas and Saiz 2023).

These findings reinforce the theoretical model, highlighting the role of CT in fostering metacognition, which subsequently influences socio-emotional domains. Given this empirical foundation, further examinations of the theoretical and conceptual framework of the CT–metacognition relationship is warranted to refine its explanatory scope.

The theoretical and empirical relationship between CT and metacognition is complex and subject to debate (Arslan 2018; Facione 1990; Halpern 1998; Magno 2010; Rivas and Saiz 2023). Our model posits that CT influences MC, which in turn impacts empathy and psychological well-being (PWB). This position is supported by three key arguments.

First, the dual nature of metacognition must be considered. Metacognition encompasses (1) the awareness of one’s cognitive processes and (2) the ability to plan, regulate, and allocate cognitive resources to achieve goals (Flavell 1979; Schraw and Dennison 1994). Awareness of one’s inner cognitive experiences develops through introspective practice, which requires intentionality. Problem-solving, for instance, demands introspection, particularly in constructing causal scenarios and engaging in counterfactual thinking (Kahneman 2013; Kahneman and Tversky 1982; Saiz 2020; Tversky and Kahneman 1974). These cognitive abilities, essential for effective reasoning, are enabled by CT, particularly inferential skills, which allow for individuals to generate and assess plausible scenarios (Baron 2023; Ennis 2015; Facione 1990).

In its second dimension, metacognition involves the awareness of strategies for addressing problems, encompassing organization, planning, and regulation. These metacognitive processes rely on prior CT competencies, both inferential and executive, including decision-making and problem-solving strategies (Halpern and Dunn 2021; Rivas and Saiz 2023). Metacognitive self-regulation provides clarity on pre- and post-decisional mechanisms, but cannot function independently of CT skills.

Second, evidence from CT training programs supports the functional dependence of metacognition on CT. These programs integrate cognitive skills, dispositions, and metacognitive strategies, highlighting how CT employs metacognition components as strategic tools for its development. The meta-analysis by Abrami et al. (2015) found that CT interventions enhance both analytical skills and metacognitive processes, such as self-monitoring. Similarly, Marin and Halpern (2011) reported that explicit CT instruction improves decision-making and critical analysis by incorporating reflective practices linked to metacognition. This suggests that, in educational contexts, metacognition develops as an integral part of CT rather than as an independent precursor or end goal. Enhancing CT skills increases awareness of cognitive processes, which in turn accelerates CT development, reinforcing a cyclical, rather than sequential, relationship. Without CT competencies, metacognition could not fully emerge.

Third, CT has been conceptualized as a theory of action, emphasizing problem-solving over isolated cognitive skills (Baron 2023; Facione 1990; Halpern and Dunn 2021; Saiz 2020). Effective problem resolution requires activating metacognitive processes that, combined with cognitive skills and dispositions, increase the likelihood of achieving optimal solutions. Individuals with high metacognition but insufficient analytical skills or dispositions may struggle to apply effective strategies, highlighting the necessity of integrating cognitive and non-cognitive components within CT. This perspective positions CT as an overarching construct that encompasses metacognition rather than a subordinate function.

From a theoretical standpoint, reversing the model’s directionality would generate inconsistencies. If metacognition were posited as influencing CT, the role of CT in socio-emotional constructs such as empathy and psychological well-being (PWB) would be obscured. This inversion would weaken the causal link between CT and socio-emotional development, making it more difficult to explain how CT fosters these dimensions. Conversely, proposing CT as antecedent to MC maintains a coherent structure that allows for explorations of CT’s indirect effects on non-cognitive domains, although empirical validation remains necessary.

In summary, prioritizing CT → MC is justified by empirical evidence from CT training programs and by theoretical consistency integrating cognitive skills, metacognitive strategies, and dispositions. While bidirectional interactions are not ruled out, establishing CT as a precursor to metacognition strengthens the model’s validity and opens pathways for educational applications, particularly in designing interventions that make metacognitive strategies explicit. This approach, while promising, highlights the need for further research, especially on the intersection of CT with well-being and empathy, an area still underexplored in the literature.

However, this study has limitations. First, the sample size may restrict the generalizability of the findings, as small samples can affect parameter stability and introduce bias (Kretzschmar and Gignac 2019). Future studies should replicate these results with larger and more diverse samples to improve external validity. Additionally, the correlational design limits causal inferences. Although structural equation modeling reveals significant associations, longitudinal studies are needed to examine how these relationships evolve over time and to clarify the direction of effects.

Another limitation of this study involves its reliance on self-report measures, which may be influenced by social desirability and subjective biases. Although objective assessments, such as PENCRISAL, were used to mitigate these biases, future research should adopt multi-method approaches combining self-reports, behavioral observations, and performance evaluations for a more comprehensive understanding of the constructs.

In conclusion, this study provides empirical evidence of the influence of CT on the enhancement of metacognitive processes and their significant associations with PWB and empathy. The findings advocate for integrating CT and metacognition into education to foster cognitive and socio-emotional growth.

## Figures and Tables

**Table 1:** Correlations of variables.

–	1	2	3	4	5	6	7	8	9	10	11	12	13	14	15	16	17	18	19	20	21	22	23
1. CT	–	0.76 **	0.62 **	0.65 **	0.65 **	0.64 **	0.11	0.05	−0.07	0.27 **	0.26 **	0.23 **	0.16	0.15	0.15	0.28 **	0.13	0.23 **	0.16 *	0.21 **	0.21 **	0.20 *	0.20 *
2. DR	0.76 **	–	0.35 **	0.36 **	0.40 **	0.37 **	0.12	0.07	−0.04	0.24 **	0.20 *	0.22 **	0.08	0.12	0.14	0.18 *	0.09	0.25 **	0.08	0.22 **	0.21 **	0.21 **	0.20 *
3. IR	0.62 **	0.35 **	–	0.36 **	0.23 **	0.28 **	0.06	0.03	−0.07	0.17 *	0.24 **	0.15	0.17 *	0.20 *	0.13	0.26 **	0.18 *	0.09	0.16 *	0.16	0.19 *	0.14	0.11
4. PR	0.65 **	0.36 **	0.36 **	–	0.24 **	0.18 *	0.05	0.05	−0.04	0.10	0.11	0.07	0.11	0.00	0.07	0.19 *	0.03	0.12	0.05	0.11	0.07	0.10	0.14
5. DM	0.65 **	0.40 **	0.23 **	0.24 **	–	0.25 **	0.03	−0.05	−0.10	0.25 **	0.09	0.09	0.07	−0.03	0.00	0.17 *	0.04	0.15	0.04	0.00	0.01	−0.01	−0.01
6. PS	0.64 **	0.37 **	0.28 **	0.18 *	0.25 **	–	0.10	0.07	0.00	0.15	0.23 **	0.21 **	0.11	0.23 **	0.16	0.14	0.13	0.12	0.21 **	0.22 **	0.21 **	0.21 *	0.20 *
7. EMP	0.11	0.12	0.06	0.05	0.03	0.10	–	0.86 **	0.65 **	0.59 **	0.36 **	0.32 **	0.15	0.35 **	0.25 **	0.29 **	0.29 **	0.23 **	0.18 *	0.29 **	0.25 **	0.28 **	0.29 **
8. CE	0.05	0.07	0.03	0.05	−0.05	0.07	0.86 **	–	0.36 **	0.26 **	0.23 **	0.18 *	0.05	0.24 **	0.16	0.22 **	0.19 *	0.09	0.11	0.17 *	0.13	0.15	0.20 *
9. EE	−0.07	−0.04	−0.07	−0.04	−0.10	0.00	0.65 **	0.36 **	–	0.15	0.26 **	0.24 **	0.11	0.11	0.19*	0.19 *	0.27 **	0.22 **	0.15	0.14	0.08	0.15	0.16 *
10. SS	0.27 **	0.24 **	0.17 *	0.10	0.25 **	0.15	0.59 **	0.26 **	0.15	–	0.34 **	0.31 **	0.22 **	0.41 **	0.22 **	0.21 **	0.19 *	0.25 **	0.15	0.36 **	0.36 **	0.35 **	0.30 **
11. MC	0.26 **	0.20*	0.24 **	0.11	0.09	0.23 **	0.36 **	0.23 **	0.26 **	0.34 **	–	0.66 **	0.69 **	0.72 **	0.77 **	0.76 **	0.77 **	0.61 **	0.67 **	0.59 **	0.54 **	0.59 **	0.54 **
12. DK	0.23 **	0.22 **	0.15	0.07	0.09	0.21 **	0.32 **	0.18 *	0.24 **	0.31 **	0.66 **	–	0.49 **	0.56 **	0.35 **	0.46 **	0.32 **	0.46 **	0.21 **	0.57 **	0.52 **	0.56 **	0.52 **
13. PK	0.16	0.08	0.17 *	0.11	0.07	0.11	0.15	0.05	0.11	0.22 **	0.69 **	0.49 **	–	0.62 **	0.38 **	0.46 **	0.45 **	0.40 **	0.36 **	0.40 **	0.37 **	0.40 **	0.35 **
14. CK	0.15	0.12	0.20 *	0.00	−0.03	0.23 **	0.35 **	0.24 **	0.11	0.41 **	0.72 **	0.56 **	0.62 **	–	0.39 **	0.40 **	0.48 **	0.39 **	0.40 **	0.56 **	0.51 **	0.57 **	0.51 **
15. P	0.15	0.14	0.13	0.07	0.00	0.16	0.25 **	0.16	0.19 *	0.22 **	0.77 **	0.35 **	0.38 **	0.39 **	–	0.51 **	0.65 **	0.33 **	0.60 **	0.43 **	0.37 **	0.44 **	0.41 **
16. O	0.28 **	0.18 *	0.26 **	0.19 *	0.17 *	0.14	0.29 **	0.22 **	0.19*	0.21 **	0.76 **	0.46 **	0.46 **	0.40 **	0.51 **	–	0.47 **	0.49 **	0.39 **	0.38 **	0.39 **	0.33 **	0.34 **
17. M	0.13	0.09	0.18*	0.03	0.04	0.13	0.29 **	0.19 *	0.27 **	0.19*	0.77 **	0.32 **	0.45 **	0.48 **	0.65 **	0.47 **	–	0.36 **	0.58 **	0.33 **	0.27 **	0.36 **	0.30 **
18. DS	0.23 **	0.25 **	0.09	0.12	0.15	0.12	0.23 **	0.09	0.22 **	0.25 **	0.61 **	0.46 **	0.40 **	0.39 **	0.33 **	0.49 **	0.36 **	–	0.21 **	0.36 **	0.36 **	0.34 **	0.30 **
19. E	0.16 *	0.08	0.16 *	0.05	0.04	0.21 **	0.18 *	0.11	0.15	0.15	0.67 **	0.21 **	0.36 **	0.40 **	0.60 **	0.39 **	0.58 **	0.21 **	–	0.37 **	0.30 **	0.37 **	0.36 **
20. PWB	0.21 **	0.22 **	0.16	0.11	0.00	0.22 **	0.29 **	0.17*	0.14	0.36 **	0.59 **	0.57 **	0.40 **	0.56 **	0.43 **	0.38 **	0.33 **	0.36 **	0.37 **	–	0.94 **	0.93 **	0.93 **
21. SA	0.21 **	0.21 **	0.19 *	0.07	0.01	0.21 **	0.25 **	0.13	0.08	0.36 **	0.54 **	0.52 **	0.37 **	0.51 **	0.37 **	0.39 **	0.27 **	0.36 **	0.30 **	0.94 **	–	0.82 **	0.80 **
22. EM	0.20 *	0.21 **	0.14	0.10	−0.01	0.21 *	0.28 **	0.15	0.15	0.35 **	0.59 **	0.56 **	0.40 **	0.57 **	0.44 **	0.33 **	0.36 **	0.34 **	0.37 **	0.93 **	0.82 **	–	0.82 **
23. PL	0.20 *	0.20 *	0.11	0.14	−0.01	0.20 *	0.29 **	0.20*	0.16 *	0.30 **	0.54 **	0.52 **	0.35 **	0.51 **	0.41 **	0.34 **	0.30 **	0.30 **	0.36 **	0.93 **	0.80 **	0.82 **	–

Note. ** *p* < .01; * *p* < .05. CT: critical thinking, DR: deductive reasoning, IR: inductive reasoning, PR: practical reasoning, DM: decision-making, PS: problem-solving; EMP: empathy, CE: cognitive empathy, EE: emotional reactivity, SS: social skills; MC: metacognition, DK: declarative knowledge, PK: procedural knowledge, CK: conditional knowledge, P: planning, O: organization, M: monitoring, DS: debugging strategies, E: evaluation; PWB: psychological well-being, SA: self-acceptance, EM: environmental mastery, PL: purpose in life.

**Table 2:** Structural model paths.

Path	*b*	*SE*	95% CI	*t*	*β*	*p*
MC ← CT	0.67	0.23	[0.22, 1.12]	2.99	0.35	.003
PWB ← MC	1.54	0.20	[1.15, 1.93]	7.62	0.71	<.001
EMP ← MC	0.39	0.09	[0.21, 0.57]	4.38	0.64	<.001

## Data Availability

Data are contained within the article.
